# Predictive value of the neutrophil-to-lymphocyte ratio for treatment response in patients diagnosed with definite or probable autoimmune encephalitis/encephalopathy

**DOI:** 10.3389/fneur.2023.1284717

**Published:** 2023-10-23

**Authors:** Shuhei Ogami, Jinsoo Koh, Katsuichi Miyamoto, Megumi Mori, Maiko Takahashi, Yoshiaki Nakayama, Mayumi Sakata, Yasuhiro Hiwatani, Yoshinori Kajimoto, Hiroshi Ishiguchi, Hidefumi Ito

**Affiliations:** ^1^Department of Neurology, Wakayama Medical University, Wakayama, Japan; ^2^Department of Neurology, Wakayama Rosai Hospital, Wakayama, Japan; ^3^Department of Neurology, Shingu Municipal Medical Center, Wakayama, Japan

**Keywords:** neutrophils, neutrophil-to-lymphocyte ratio, neutrophil extracellular traps, short-term treatment response, definite or probable autoimmune encephalitis/encephalopathy

## Abstract

**Introduction:**

Autoimmune encephalitis/encephalopathy (AE) is a complex and heterogeneous disease, making it difficult to predict the prognosis. The neutrophil-to-lymphocyte ratio (NLR) has emerged as a potential prognostic tool, but its usefulness remains a matter of debate. This study aimed to explore prognostic factors in cases of clinically definite or probable AE, including those with autoantibody-negative, or unknown status.

**Methods:**

Data on patients diagnosed with definite or probable AE, including those with autoantibody-negative, or unknown status, were retrospectively collected from the admission records of our department between January 2013 and December 2022. These patients were then categorized into either a good- or poor-response group, based on their short-term treatment response. Clinical characteristics, auxiliary examinations, and treatments were compared between the two groups. A multivariable logistic regression model was constructed to identify independent predictors of poor short-term treatment response by Akaike information criterion backward stepwise method.

**Results:**

A total of 31 patients were included in the final analysis, with 18 of them included in the poor-response group. In the univariable analysis, the poor-response group had a higher proportion of patients with a modified Rankin Scale (mRS) high score upon admission, female, epileptic seizures, or NLRs of 3.93 or higher than the good-response group (all *p* < 0.10). Furthermore, the multivariable logistic regression analysis revealed that the mRS score upon admission [OR: 5.51, 95% confidence intervals (CI): 1.29–23.50, *p* = 0.02], epileptic seizures (OR: 10.01, 95% CI: 1.16–86.66, *p* = 0.04), and NLRs of 3.93 or higher (OR: 11.37, 95% CI: 1.12–114.68, *p* = 0.04) were significantly associated with poor short-term treatment response.

**Conclusion:**

The NLR may play a supplementary role in predicting the short-term treatment response in patients diagnosed with definite or probable AE, including those with autoantibody-negative, or unknown status.

## Introduction

Acute encephalitis is an inflammatory disorder of the brain. Encephalitis/encephalopathy with an autoimmune mechanism is referred to as autoimmune encephalitis/encephalopathy (AE). The incidence of AE in high-income countries is approximately 5–10 per 100,000 people per year (1). However, the incidence is increasing due to the identification of new autoantibodies and growing awareness of this disease (2). A previous study demonstrated that anti-N-methyl-D-aspartate receptor (anti-NMDAR) encephalitis was diagnosed more frequently (four times) than viral encephalitis in patients aged 30 years or younger (3). Autoimmunity has been recognized as one of the major causes of encephalitis or encephalopathy.

The prognosis for AE is generally poor. However, anti-NMDAR encephalitis has a better prognosis than other forms of AE, with up to 80% of patients achieving a modified Rankin Scale (mRS) score of 0–2 within 24 months (2). Nevertheless, a recent study found that only 61% of patients were able to return to their previous work or school life (4). As a result, diagnostic criteria for early detection of AE have been established (1), and numerous studies have investigated potential prognostic factors for AE. Factors such as age, mRS score upon admission, delay in initiating immunotherapy, cerebrospinal fluid (CSF) examination, and others have been investigated, but the results remain controversial (5–7). Recently, the neutrophil count has also been suggested as a potential prognostic factor for AE. The plasma neutrophil-to-lymphocyte ratio (NLR), calculated by dividing the absolute count of neutrophils by the absolute count of lymphocytes, has been proposed as a marker for systemic inflammation and stress (8). NLR is a simple indicator that can be easily evaluated in any hospital setting. Previous studies have shown that an elevated NLR is associated with poor treatment response and prognosis in patients with autoantibody-positive AE (9–11).

However, measuring various types of autoantibodies can often be challenging due to limitations such as facility availability, technical skills, sample volumes, and the health insurance system. As a result, some patients are tentatively diagnosed with AE as autoantibody-negative or with an unknown autoantibody status. To the best of our knowledge, there have been few studies investigating the association between the NLR and the prognosis of cases of definite or probable AE, including those with autoantibody-negative, or unknown status. One study reported the potential association between the NLR and prognosis in a group of AE cases, including those with autoantibody-negative, or unknown status (12). However, it is worth noting that this study included a significant number of patients with possible AE. The diagnostic criteria for AE recommend reconsidering the diagnosis if autoantibodies are negative and patients do not fulfill the criteria for probable autoimmune encephalitis (1). Therefore, whether the NLR can serve as a prognostic factor in cases of definite or probable AE, including those with autoantibody-negative, or unknown status, is still unclear, and requires further assessment.

We hypothesized that the NLR could be a potential prognostic factor in cases of definite or probable AE, including those with autoantibody-negative, or unknown status. To investigate this, we conducted this study utilizing data from the admission records of our department. The primary objective of this study was to assess the association between the NLR and treatment response in cases of definite or probable AE, including those with autoantibody-negative, or unknown status. In addition, we aimed to investigate the association between clinical features and prognostic factors within the same group of patients.

## Materials and methods

### Ethical approval

This retrospective cohort study was approved by the Institutional Review Board of Wakayama Medical University (Approval Number 3788). Informed consent was obtained through an opt-out disclosure process.

### Study design and patients

In this study, candidate patients were recruited from the admission records of our department between January 2013 and December 2022. Our search strategy involved using keywords such as “limbic,” “encephalitis,” “encephalopathy,” “encephalomyelitis,” “leukoencephalopathy,” or “paraneoplastic”. Patients who were aged 16 years, met the diagnostic criteria for definite or probable AE (1), and had an initial diagnosis received immunotherapy or underwent tumor resection were included in this study. In the initial diagnostic assessment, patients who met the diagnostic criteria for possible AE upon admission were recruited. A diagnosis of possible AE was made based on the following criteria: subacute onset (<3 months) of working memory deficits (short-term memory loss), altered mental status, or psychiatric symptoms and at least one of the following conditions: new focal central nervous system (CNS) findings, seizures that could not be explained by a previously known seizure disorder, CSF abnormalities [white blood cell (WBC) count of >5 cells per mm^3^], or magnetic resonance imaging (MRI) abnormalities indicative of encephalitis. Subsequently, patients who tested positive for autoantibodies were diagnosed with definite AE. Patients who did not test positive for autoantibodies and who were not diagnosed with other CNS disorders were evaluated to confirm if they met the diagnostic criteria for definite autoimmune limbic encephalitis or probable AE. Patients were diagnosed with definite autoimmune limbic encephalitis if they met the following criteria: subacute onset (<3 months) of working memory deficits, seizures, or psychiatric symptoms indicating the involvement of the limbic system, T2-weighted fluid-attenuated inversion recovery MRI abnormalities (bilateral brain abnormalities highly restricted to the medial temporal lobes), and at least one of the following conditions: CSF abnormalities (WBC count of >5 cells per mm^3^) or electroencephalogram (EEG) abnormalities (epileptic or slow-wave activity involving the temporal lobes). A diagnosis of probable AE was made if the following criteria were met: rapid progression (<3 months) of working memory deficits (short-term memory loss), altered mental status, or psychiatric symptoms; exclusion of well-defined syndromes of autoimmune encephalitis; and at least two of the following conditions: MRI abnormalities indicative of autoimmune encephalitis, CSF abnormalities (pleocytosis, CSF-specific oligoclonal bands or an elevated CSF IgG index, or both), or brain biopsy showing inflammatory infiltrates and excluding other disorders. Patients were excluded if they tested positive for the anti-aquaporin 4 antibody, were diagnosed with neuromyelitis optica spectrum disorders, were lost to follow-up, had incomplete clinical data, or had other CNS disorders. In addition, two clinical neurology specialists, Shuhei Ogami, and Jinsoo Koh, carefully reviewed the medical records of the patients to determine whether they met the criteria for AE with autoantibody-negative or unknown status (definite autoimmune limbic encephalitis or probable AE) and made decisions regarding their inclusion in the study.

### Data collection

Data on demographics, including gender, and age, along with neurological disease severity upon admission, clinical symptoms, and features, disease course, laboratory findings, CSF examination findings, EEG, brain MRI findings, and treatments were collected from the patients' medical records. The medical records were reviewed from admission until 4 weeks after the initiation of immunotherapy or tumor resection. The severity of neurological disease was assessed using the mRS score (13). Clinical symptoms and features included loss of consciousness, memory loss, behavioral changes, a fever of 38°C or higher, epileptic seizures, psychiatric symptoms, movement disorders, autonomic dysfunction, and tumor complications. Laboratory tests included WBC, neutrophil, and lymphocyte counts, as well as NLRs. CSF analysis involved assessing protein levels and cell counts. The initial laboratory and CSF examination findings were retrieved from admission until the initiation of immunotherapy or tumor resection. Treatments included intravenous methylprednisolone pulse (500–1,000 mg) for 3 days, intravenous gamma immunoglobulin (400 mg/kg) for 5 days, plasma exchange, cyclophosphamide, tumor resection, oral prednisolone, antiepileptic drugs, and mechanical ventilation, either alone, or in combination. Patients were categorized into a good- or poor-response group based on their short-term treatment response, as previously reported (14). The good response was defined as sustained improvement and a mRS score of 3 or lower at 4 weeks after the initiation of immunotherapy or tumor resection. The poor response was defined as either the absence of sustained improvement or a sustained mRS score of 4 or higher within 4 weeks after the initiation of immunotherapy or tumor resection.

### Statistical analysis

The statistical analysis was conducted using JMP Pro 14.1.0. A two-sided *p*-value of <0.05 was considered statistically significant. Continuous variables were presented as means and standard deviations or medians and interquartile ranges based on their distribution. The Shapiro–Wilk test was used to check their distribution. Continuous variables were categorized by the mean or median value of all patients based on their distribution. Categorical variables were expressed as counts and percentages. The student's *t*-test or Mann–Whitney *U*-test was used to compare continuous variables, while Fisher's exact test was used to compare categorical variables between the two groups. A multivariable logistic regression model was constructed to assess the association between independent variables and poor short-term treatment response. Clinical features and auxiliary examinations in categorized variables with *p*-values < 0.10 in the univariable analysis were chosen as candidate variables. The Akaike information criterion (AIC) backward stepwise method was used to select the potential independent predictors. However, a mRS score in a continuous variable was defined as the coefficient variable, and was forced to be included in the multivariable logistic regression model when backward stepwise method was used. Odds ratios (OR) and 95% confidence intervals (CI) were used to quantify the strength of these associations. The receiver operating characteristic (ROC) curve was used to estimate the cutoff value for the NLR and short-term treatment response.

## Results

### Clinical characteristics

**Figure 1:**
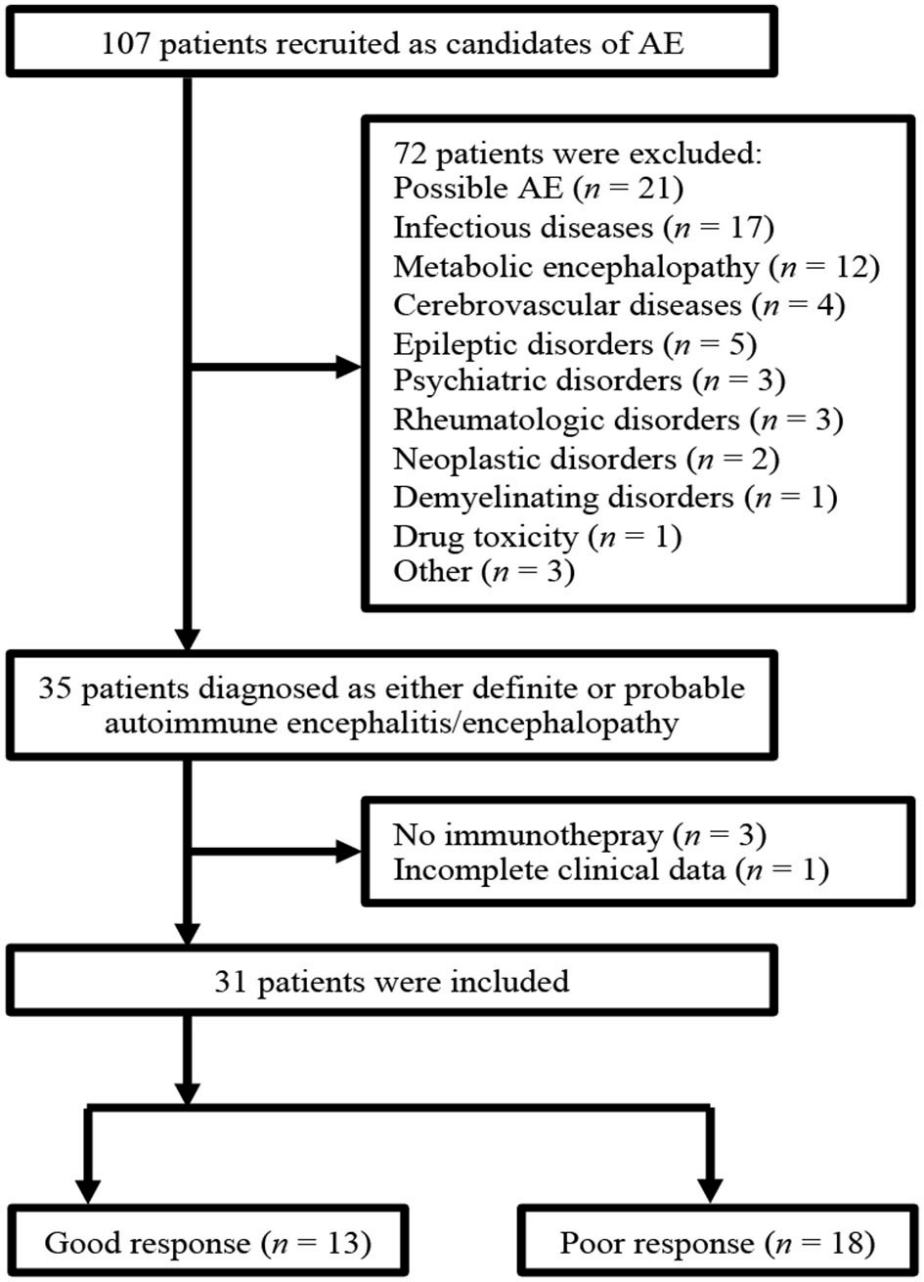
The flowchart of patient selection. NMOSD, neuromyelitis optica spectrum disorders.

Figure 1 shows the flowchart of patient selection. A total of 107 patients met the diagnostic criteria for possible AE upon admission. Among them, 72 patients were excluded because they were finally diagnosed with possible AE or other CNS disorders. Thirty-five patients fulfilled the diagnostic criteria for either definite AE, definite autoimmune limbic encephalitis, and probable AE. Four patients were excluded from this group for various reasons. Three patients showed improvement before initiating immunotherapy and therefore did not receive it, while one patient had incomplete clinical data. Consequently, 31 patients were included in the final analysis. Out of these, 13 patients were classified into the good-response group, while 18 patients fell into the poor-response group. Further details regarding the final diagnosis are provided in the Supplementary Table 1. Notably, all patients with definite autoimmune limbic encephalitis tested negative for the anti-NMDAR antibody.

Table 1 summarizes the clinical characteristics of the patients. The mRS score upon admission were significantly higher in the poor-response group than in the good-response group. Furthermore, the poor-response group had a significantly higher proportion of patients with epileptic seizures than the good-response group. However, no significant differences were observed in other variables between the two groups. There were a total of 10 cases with tumor complications. Specifically, there were four cases of mature cystic teratoma and one case of small-cell lung carcinoma in anti-NMDAR encephalitis. In autoimmune glial fibrillary acidic protein astrocytopathy, there was one case of mucinous cystic neoplasm and another case of ovarian teratoma. In addition, there was one case of a retroperitoneal tumor in anti-glutamate receptor encephalitis, one case of papillary renal cell carcinoma in anti-CV2 antibody-associated encephalitis, and one case of lung squamous cell carcinoma in anti-Hu and anti-amphiphysin antibody-associated encephalitis.

**Table 1:** Demographic and clinical characteristics.

	**All (*n* = 31)**	**Good-response group (*n* = 13)**	**Poor-response group (*n* = 18)**	***p*-value**
Age, years	52 ± 20	53 ± 20	52 ± 21	0.88
Age above the mean value, years, *n* (%)	15 (48.4)	7 (53.9)	8 (44.4)	0.61
Female, *n* (%)	14 (45.2)	3 (23.1)	11 (61.1)	0.07
mRS score upon admission	5 (4–5)	4 (3–5)	5 (5–5)	0.01
Tumor complications, *n* (%)	10 (32.3)	2 (15.4)	8 (44.4)	0.13
Latency from symptom onset to treatment, days	11 (7–41)	15 (7–54)	10 (7–31)	0.43
**Symptoms**, ***n*** **(%)**				
Loss of consciousness	29 (93.5)	13 (100)	16 (88.9)	0.50
Memory loss	23 (74.2)	11 (84.6)	12 (66.7)	0.41
Behavioral changes	20 (64.5)	8 (61.5)	12 (66.7)	1.00
Fever (38°C or above)	17 (54.8)	6 (46.2)	11 (61.1)	0.48
Epileptic seizures	17 (54.8)	4 (30.8)	13 (72.2)	0.03
Psychiatric symptoms	15 (48.4)	7 (53.9)	8 (48.4)	0.72
Movement disorders	9 (29.0)	3 (23.1)	6 (33.3)	0.70
Autonomic dysfunction	7 (22.6)	2 (15.4)	5 (27.8)	0.67

mRS, modified Rankin Scale.

### Laboratory examinations and treatments

Laboratory, CSF examination, EEG, and brain MRI findings are summarized in Table 2. The median NLR was 3.93, and the proportion of patients with NLRs of 3.93 or higher tended to be higher in the poor-response group than in the good-response group, but the difference was not statistically significant. No other variables also showed statistically significant differences.

**Table 2:** Auxiliary examination data.

	**All (*n* = 31)**	**Good-response group (*n* = 13)**	**Poor-response group (*n* = 18)**	***p*-value**
**Laboratory tests**, ***n*** **(%)**				
WBCs × 10^3^/μL	8.09 (4.96–103.40)	7.53 (4.75–9.43)	8.76 (5.29–113.50)	0.19
Neutrophils × 10^3^/μL	5.82 (2.86–8.46)	5.00 (2.71–7.11)	7.23 (3.52–100.60)	0.15
Lymphocytes × 10^3^/μL	1.42 (0.98–1.72)	1.43 (0.96–2.25)	1.36 (0.98–1.52)	0.45
NLR	3.93 (2.44–6.02)	3.22 (2.12–4.79)	5.07 (2.47–8.49)	0.06
NLRs at or above the median value, *n* (%)	16 (51.6)	4 (30.8)	12 (66.7)	0.07
**CSF examination**				
Protein, mg/dL	53 (31–100)	60 (40–130)	49 (30–78)	0.22
Cell counts, /μL	15 (6–41)	28 (9–62)	13 (6–35)	0.20
**EEG data**, ***n*** **(%)**				
Slow-wave	26 (83.9)	11 (84.6)	15 (83.3)	1.00
ESz or ESE	4 (12.9)	0 (0.0)	4 (22.2)	0.12
**MRI findings**, ***n*** **(%)**				
FLAIR lesions	23 (74.2)	9 (69.2)	14 (77.8)	0.69
DWI lesions	7 (22.6)	3 (23.1)	4 (22.2)	1.00

WBCs, white blood cells; NLR, neutrophil-to-lymphocyte ratio; CSF, cerebrospinal fluid; EEG, electroencephalogram; ESz, electrographic seizure; ESE, electrographic status epilepticus; MRI, magnetic resonance imaging.

There were no significant differences in terms of immunotherapy between the two groups (Table 3). However, 29 patients (93.5%) received methylprednisolone pulse initially, while none received cyclophosphamide until 4 weeks after the initiation of immunotherapy or tumor resection. All four patients who received tumor resection had mature cystic teratoma with anti-NMDAR encephalitis. There was no significant difference in terms of antiepileptic drug induction between the two groups.

**Table 3:** Patients' treatments.

	**All (*n* = 31)**	**Good-response group (*n* = 13)**	**Poor-response group (*n* = 18)**	***p*-value**
**First-line therapy**				
Methylprednisolone pulse, *n* (%)	30 (96.8)	13 (100)	17 (94.4)	1.00
IVIg, *n* (%)	19 (61.3)	7 (53.9)	12 (66.7)	0.71
Plasma exchange, *n* (%)	3 (9.7)	0 (0.0)	3 (16.7)	0.25
**Other therapy**				
Tumor resection, *n* (%)	4 (12.9)	0 (0.0)	4 (22.2)	0.12
Prednisolone, *n* (%)	13 (41.9)	8 (61.5)	5 (27.8)	0.08
Prednisolone initiation dose (mg/kg)	0.8 (0.70–1.00)	0.78 (0.72–0.82)	1.00 (0.60–1.00)	0.42
Antiepileptic drugs, *n* (%)	18 (58.0)	6 (46.2)	12 (66.7)	0.29
Mechanical ventilation and ICU stay, *n* (%)	7 (22.6)	0 (0.0)	7 (38.9)	0.02

IVIg, intravenous gamma immunoglobulin; ICU, intensive care unit.

### Variables associated with poor short-term treatment response

To identify independent predictors for poor short-term treatment response, the mRS score upon admission and categorical variables with *p*-values < 0.10 (female, epileptic seizures, and NLRs of 3.93 or higher) were included in the AIC backward stepwise method. The AIC backward stepwise method selected epileptic seizures, and NLRs of 3.93 or higher as the final independent predictors in addition to the mRS score upon admission. The multivariable logistic regression analysis revealed that the mRS score upon admission (OR: 5.51, 95% CI: 1.29–23.50, *p* = 0.02), epileptic seizures (OR: 10.01, 95% CI: 1.16–86.66, *p* = 0.04), and NLRs of 3.93 or higher (OR: 11.37, 95% CI: 1.12–114.68, *p* = 0.04) were significantly associated with poor short-term treatment response (Table 4).

**Table 4:** Variables associated with a poor response to treatments.

	**OR (95% CI)**	***p*-value**
An mRS score upon admission	5.51 (1.29–23.50)	0.02
Epileptic seizure	10.01 (1.16–86.66)	0.04
NLRs of 3.93 or higher	11.37 (1.12–114.68)	0.04

OR, odds ratio; CI, confidence interval; mRS, modified Rankin Scale; NLR, neutrophil-to-lymphocyte ratio.

An mRS score upon admission in a continuous variable was defined as the coefficient variable, and was forced to be included in the multivariable logistic regression model. Other socio-demographic factors were not taken into account because of small sample size.

**Figure 2:**
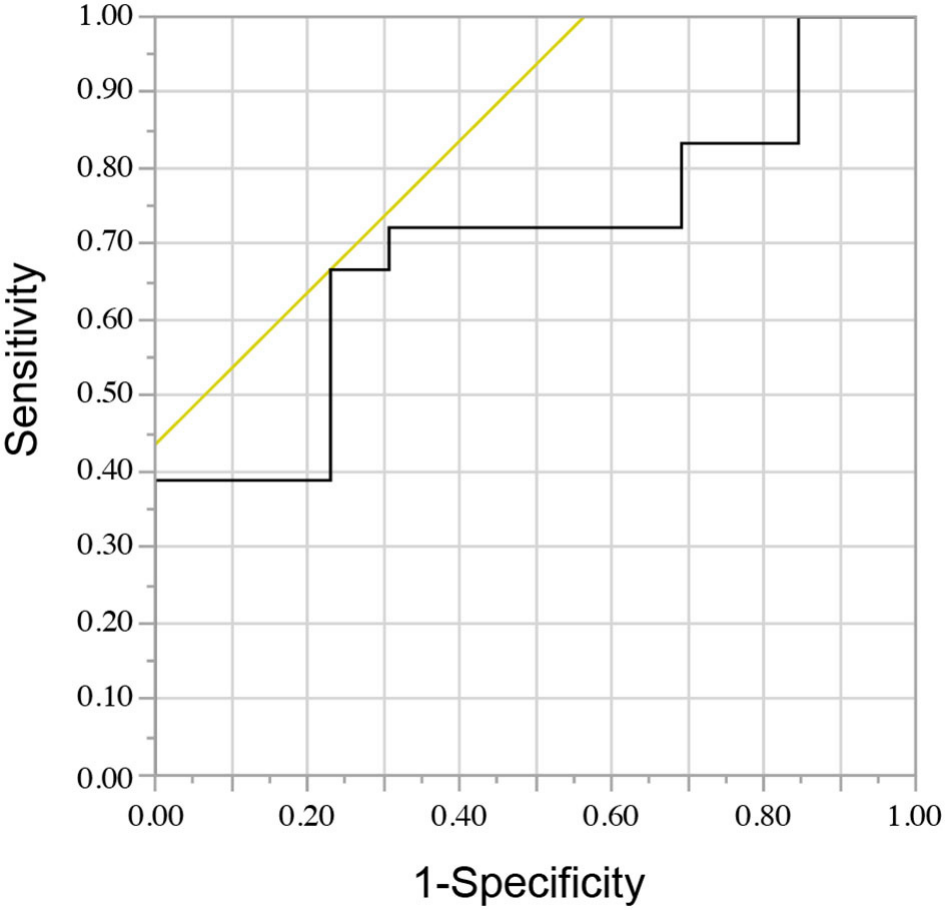
The receiver operating characteristic curve for the neutrophil-to-lymphocyte ratio and short-term treatment response.

Figure 2 shows the ROC curve for the NLR and short-term treatment response. The area under the curve was 0.70, with a sensitivity of 0.67 and a specificity of 0.69 when the median NLR value of 3.93 was set as the cutoff. Using Youden's index to estimate the optimal cutoff value, it was determined to be 4.10, with an area under the curve of 0.70, a sensitivity of 0.67, and a specificity of 0.77.

## Discussion

Our study demonstrated that the NLR can be used to predict short-term treatment response in patients diagnosed with definite or probable AE, including those with autoantibody-negative, or unknown status. The multivariable logistic regression model revealed a significant association between NLRs of 3.93 or higher and poor short-term treatment response. In clinical practice, there is often a need to make a tentative diagnosis and predict the prognosis of AE, particularly when specific autoantibodies are not immediately or fully identified. A previous study highlighted the relationship between NLR and treatment response in patients with AE, including those with autoantibody-negative, or unknown status (12). However, this study included cases of possible AE, potentially encompassing conditions other than AE, which limits the ability to establish a definitive relationship between NLR and AE prognosis. In our study, we observed similar results, particularly in patients diagnosed with definite or probable AE, suggesting that measuring NLR can be used to predict treatment response, even in patients who tentatively meet the diagnostic criteria for definite or probable AE.

A previous study showed that a high NLR was associated with poor prognosis during the final follow-up (15). However, in this study, there was no significant association between NLR and failed first-line treatment. One possible reason is the definition of first-line treatment response. A previous study defined clinical response as improvement in mRS score to ≥1 after 2–4 weeks of the first-line treatment. However, this definition is different from ours. Another potential reason is disease severity. A previous study only included patients with severe autoantibody-positive AE who required mechanical ventilation or admission to the intensive care unit. All patients with severe condition in our study were classified under the poor-response group (Table 3). Patients with severe AE may present with minimal short-term improvement after the first-line treatment, potentially obscuring the statistical difference between the two groups in the previous study.

An optimal NLR cutoff value of 4.10 was considered for predicting poor short-term treatment response (Figure 2). Another report, which included cases of AE with autoantibody-negative or unknown status, revealed that an NLR cutoff value of 4.45 could be used to predict adverse outcomes (12). Taking these factors into consideration, a cutoff value of approximately 4 may indicate a poor prognosis. However, further studies should be conducted to confirm the reproducibility of these findings.

The pathophysiological mechanism related to the association between neutrophils and AE remains uncertain. However, some studies have revealed that neutrophil extracellular traps (NETs) play a role in inflammation in autoimmune diseases (16). If neutrophils are exposed to various stimuli, they release NETs, which activate B cells and macrophages, leading to the production of autoantibodies and the secretion of interleukin (IL)-8, IL-6, and tumor necrosis factor-alpha (TNF-α) (17). Enzymes within NETs can also be a source of autoantigens (18). Neutrophils and NETs may also be associated with multiple sclerosis (MS), an autoimmune disease of the CNS. MS is characterized by immune-associated demyelination of the CNS, resulting in neuronal loss and substantial disability (19). Patients with MS have a high neutrophil count and NET formation marker level (19, 20). In addition, NET-associated proteins can damage the blood–brain barrier in MS (20). Neutrophils may contribute to the pathology of CNS diseases by generating NETs. In a recent study on patients with anti-NMDAR encephalitis, the production of NET increased, and the NET levels were found to be correlated with the levels of IL-8, IL-6, and TNF-α (21). Neutrophils may enhance immune response via NET generation in AE pathology. Therefore, a higher neutrophil count may indicate a less effective response to immunotherapy.

Our study found a significant association between epileptic seizures and short-term treatment response in the multivariate logistic regression model. The association between epileptic seizures and prognosis remains controversial (6, 22). This conflicting result may be attributed to variations in the detection rate of epileptic seizures among different hospitals. To increase the rate of abnormality detection, it is necessary to repeat EEGs or conduct long-term EEG monitoring, as standard EEGs can only identify interictal epileptic abnormalities in 30–50% of cases (23). Our study recruited patients with epileptic seizures based on their medical history and standard EEG results. None of the patients underwent long-term EEG monitoring. Thus, we could have overlooked non-convulsive epileptic seizures in patients experiencing loss of consciousness. The lack of long-term EEG monitoring is one of the limitations of our study, thereby underscoring the need for a standardized protocol for EEG monitoring.

In our analysis, the mRS score upon admission was another prognostic factor. However, a previous study reported no association between the mRS score upon admission and prognosis (24). This conflicting result may be attributed to several reasons. One possible reason is the variation in background diseases across the studies. The previous study had a higher proportion of patients with anti-NMDAR encephalitis than our study. In the subgroup analysis, the mRS score upon admission was not associated with prognosis in patients with anti-NMDAR encephalitis but was associated with anti-leucine-rich glioma inactivated 1 encephalitis (24). Therefore, the impact of the mRS score upon admission on the prognosis may depend on the background disease. Another potential reason for this conflicting result is the reliability of the mRS score itself. While the mRS score is frequently used in clinical research, it is a subjective assessment, and a previous study has highlighted uncertainties regarding its reliability (25). Recently, a more detailed and novel assessment scale for AE called the clinical assessment scale in autoimmune encephalitis (CASE) has been proposed (26). Ideally, disease severity should be assessed using both the mRS score and CASE. However, our study was limited by incomplete clinical data, which prevented us from utilizing CASE. This limitation should be acknowledged as well.

Our study has some limitations that should be acknowledged. First, it was a retrospective study conducted at a single hospital. Second, the small sample size might have led to bias and limited statistical power. Hence, an effort was made to decrease these limitations by adopting strict criteria for selecting potential independent predictors in the multivariate logistic regression model. Continuous variables, except the mRS score, were not selected as candidate independent variables. In addition, the number of independent variables was adjusted. Hence, it was not extremely large relative to the sample size. However, coefficient variables were challenging to control due to small sample size. The number of independent variables was limited to three because the sample size was 31. Hence, we could not choose gender, age, and other variables, except the mRS score, as coefficient variables. Third, approximately half of the patients were lost to follow-up within 1 year, which hindered our analysis of the long-term prognosis and the long-term prognosis could not be analyzed. It would be beneficial to extend the follow-up period to at least 1 year, as previous studies have done. Fourth, the measured antibodies varied among patients. It is important to establish a standardized examination protocol for measuring at least commercially available autoantibodies. Lastly, due to restrictions imposed by the Japanese health insurance system, immunotherapy drugs such as rituximab, bortezomib, mycophenolate mofetil, and methotrexate were not administered. If these drugs become accessible in the future, it is possible that the results may be altered.

## Conclusion

Our study has demonstrated that an elevated NLR may be associated with poor short-term treatment response in patients diagnosed with definite or probable AE, including those with autoantibody-negative or unknown status. This suggests that neutrophils may contribute to the pathophysiological mechanism of AE. However, the exact mechanism remains unclear and requires further investigation.

## Data availability statement

The original contributions presented in the study are included in the article/Supplementary material, further inquiries can be directed to the corresponding author.

## Ethics statement

The studies involving humans were approved by the Institutional Review Board of Wakayama Medical University. The studies were conducted in accordance with the local legislation and institutional requirements. Written informed consent for participation was not required from the participants or the participants' legal guardians/next of kin because Informed consent was obtained through an opt-out disclosure process.

## Author contributions

SO: Conceptualization, Data curation, Formal analysis, Investigation, Methodology, Project administration, Writing—original draft, Writing—review and editing. JK: Conceptualization, Data curation, Formal analysis, Methodology, Project administration, Writing—original draft, Writing—review and editing. KM: Writing—review and editing, Investigation, Supervision, Funding acquisition. MM: Writing—review and editing, Investigation. MT: Investigation, Writing—review and editing. YN: Investigation, Writing—review and editing. MS: Investigation, Writing—review and editing. YH: Investigation, Writing—review and editing. YK: Investigation, Writing—review and editing. HIs: Investigation, Writing—review and editing. HIt: Writing—review and editing, Supervision.
