# Supplementary material for: Predictive value of the neutrophil-to-lymphocyte ratio for treatment response in patients diagnosed with definite or probable autoimmune encephalitis/encephalopathy
Source: Front Neurol. 2023 Oct 23;14:1284717. doi: 10.3389/fneur.2023.1284717 (PMC10626493; doi:10.3389/fneur.2023.1284717)
Supplement: Supplementary file 1 [file Table_1.DOCX]

Supplementary Material

Predictive value of the neutrophil-to-lymphocyte ratio for treatment response in patients diagnosed with definite or probable autoimmune encephalitis/encephalopathy

Shuhei Ogami^*^, Jinsoo Koh, Katsuichi Miyamoto, Megumi Mori, Maiko Takahashi, Yoshiaki Nakayama, Mayumi Sakata, Yasuhiro Hiwatani, Yoshinori Kajimoto, Hiroshi Ishiguchi, Hidefumi Ito

*** Correspondence:** Shuhei Ogami: shuogm@wakayama-med.ac.jp

# Supplementary Tables

Supplementary Table 1 The good-response group and the poor-response group.

|  | **All (*n* = 31)** | **Good-response group (*n* = 13)** | **Poor-response group (*n* = 18)** |
| --- | --- | --- | --- |
| anti-NMDAR encephalitis, *n* | 9 | 4 | 5 |
| anti-GluR encephalitis, *n* | 4 | 1 | 3 |
| autoimmune GFAP astrocytopathy, *n* | 4 | 2 | 2 |
| ADEM, *n* | 1 | 1 | 0 |
| MOGAD, *n* | 1 | 0 | 1 |
| anti-CV2 antibody-associated encephalitis, *n* | 1 | 1 | 0 |
| anti-Hu and anti-amphiphysin antibody-associated encephalitis, *n* | 1 | 0 | 1 |
| anti-VGKC complex encephalitis, *n* | 1 | 0 | 1 |
| Hashimoto encephalopathy, *n* | 1 | 1 | 0 |
| definite autoimmune limbic encephalitis, *n* | 6 | 2 | 4 |
| probable autoimmune encephalitis, *n* | 2 | 1 | 1 |

Abbreviation: N-methyl-D-aspartate-receptor (NMDAR), glutamate receptor (GluR), glial fibrillary acidic protein (GFAP), acute disseminated encephalomyelitis (ADEM), myelin oligodendrocyte glycoprotein antibody-associated disease (MOGAD), voltage-gated potassium channel complex (VGKC).
